# Beyond the Core: The Role of Supplementary Short Tandem Repeats in Forensic Genetics

**DOI:** 10.3390/ijms27135756

**Published:** 2026-06-25

**Authors:** Vitória Ramos, Benedita Ferreira-Silva, Jennifer Fadoni, António Amorim, Laura Cainé

**Affiliations:** 1 Faculty of Medicine, University of Porto, 4200-319 Porto, Portugal; mvitorianetor@gmail.com; 2 National Institute of Legal Medicine and Forensic Sciences, I.P., North Branch, 4050-202 Porto, Portugal; benedita.f.silva@inmlcf.mj.pt (B.F.-S.); jennifer.n.fadoni@inmlcf.mj.pt (J.F.); antonio.j.amorim@inmlcf.mj.pt (A.A.); 3 LAQV&REQUIMTE, Laboratory of Applied Chemistry, Department of Chemical Sciences, Faculty of Pharmacy, University of Porto, 4050-313 Porto, Portugal; 4 National Institute of Legal Medicine and Forensic Sciences, I.P., Centre Branch, 3000-548 Coimbra, Portugal; 5 Faculty of Sciences, University of Lisbon, 1749-016 Lisbon, Portugal; 6 Faculty of Medicine, University of Lisbon, 1649-028 Lisbon, Portugal

**Keywords:** short tandem repeats, forensic genetics, non-core markers, Investigator^®^ HDplex, human identification, population genetics, validation, massively parallel sequencing

## Abstract

Conventional forensic DNA profiling predominantly utilizes established core sets of autosomal short tandem repeats (STRs), such as the FBI’s Combined DNA Index System (CODIS) and the European Standard Set (ESS). While these panels are effective for standard forensic casework, they may be inadequate in more demanding scenarios, including severely degraded samples, complex multi-contributor mixtures, and kinship cases with deficiencies, where enhanced discriminatory capability is crucial. Additional non-core STR loci address these challenges while maintaining the non-coding, phenotypically uninformative nature that ensures the legal and ethical acceptability of forensic genetic evidence in court. This review assesses the forensic applications, population genetic parameters, validation requirements, and ethical considerations associated with non-core STR loci. A representative supplementary STR panel is presented as a case study to illustrate both the forensic value and the analytical requirements associated with the implementation of extended non-core STR systems. Challenges in implementation were identified in areas such as nomenclature standardization, backward compatibility with existing national databases, and geographic gaps in population reference data. The review concludes that a hybrid strategy, which retains core capillary electrophoresis (CE)-based profiling for routine casework and employs extended non-core panels for complex cases, represents the most practical path for the field.

## 1. Introduction

Forensic genetics applies molecular biology techniques and DNA markers to aid in criminal investigations, civil disputes, biological relationships, and humanitarian identification efforts [1].

Forensic DNA markers can be broadly categorized into repeat-based markers, such as short tandem repeats (STRs); sequence-based markers, including single-nucleotide polymorphisms (SNPs) and insertion/deletion polymorphisms (InDels); lineage markers, such as mitochondrial DNA (mtDNA) and X- and Y-chromosomal markers; and emerging marker systems, including microhaplotypes [2].

An important criterion in the selection of forensic STR markers is their location within non-coding regions of the genome. This minimizes the likelihood of revealing medically relevant or phenotypic information, thereby supporting the ethical principles of privacy, proportionality and data minimization that underpin forensic DNA legislation and practice in many jurisdictions [3,4].

The field has undergone tremendous evolution over the past few decades: from its origins in blood group systems and serum proteins to the introduction of polymerase chain reaction (PCR) and capillary electrophoresis (CE), and now to the omics era of high-throughput genomic investigations. Each phase has been driven by the need for greater sensitivity and discrimination [1,5,6].

Short tandem repeat typing is the current gold standard for human identification in forensic science. STRs are highly polymorphic, abundantly distributed throughout the genome, and predominantly located in non-coding intronic and intergenic regions, making them well-suited for multiplexed analysis without disclosing clinically or phenotypically sensitive genetic information [7,8]. Standardized core loci sets have been defined to standardize procedures, construct searchable national databases, and facilitate cross-jurisdictional collaboration, most notably the FBI’s Combined DNA Index System (CODIS) [9] and the European Standard Set (ESS) [10]. These panels have proven highly effective in routine casework, providing robust discriminatory power within a well-established legal and ethical framework [11,12,13].

Although core STR panels are highly effective for routine forensic casework, their performance may be challenged in situations involving degraded DNA, complex DNA mixtures, and deficiency kinship analyses [14,15,16,17,18,19]. Supplementary non-core STR loci have therefore been developed to enhance discriminatory power and evidential strength in these demanding scenarios while preserving the non-coding and phenotypically neutral nature that underpins the legal admissibility of forensic STR evidence [9,20,21].

Nonetheless, the forensic reliability and legal admissibility of additional markers necessitate thorough evaluation and rigorous validation, as well as adherence to ethical and legal standards. Moreover, the development of databases is essential to ensure accurate application and prevent errors. These requirements continue to be addressed within the field [3,4,21].

This narrative review assesses the current landscape of forensic genetic markers used in human identification, with an emphasis on non-core STRs, their applications in forensic science, their population genetic analysis, and the validation and statistical framework requirements for their proper use. Utilizing the Investigator^®^ HDplex kit (QIAGEN, Hilden, Germany) as a case study, the review underscores both the forensic advantages and the analytical challenges associated with this commercially available kit, substantiated by independent evidence from diverse global populations. Additionally, it investigates the integration of non-core STRs with massively parallel sequencing (MPS), the primary implementation challenges within the field, and the ethical and legal considerations that must be addressed when employing these markers.

Throughout this review, supplementary non-core STRs are presented not as replacements for the established core STR systems, but as complementary tools that extend the capabilities of forensic DNA analysis in challenging scenarios. The central premise is that the integration of these markers, particularly when combined with massively parallel sequencing technologies, can enhance forensic performance in complex kinship testing, degraded DNA analysis, and mixture interpretation while maintaining compatibility with existing forensic infrastructures and legal frameworks.

This narrative review was based on a structured literature search conducted in PubMed, Scopus, and Web of Science. Searches were performed using combinations of keywords including “non-core STR”, “supplementary STR”, “extended STR panel”, “forensic genetics”, “kinship analysis”, “DNA mixtures”, “massively parallel sequencing”, “microhaplotypes”, “population genetics” and related terms. The search primarily focused on articles published up to 2026. Additional relevant publications were identified through manual screening of reference lists. Literature was selected according to its relevance to the forensic applications, population genetics, technological developments, implementation challenges, and ethical and legal considerations associated with supplementary STR markers. The literature search and subsequent discussion were organized around six predefined thematic areas: (i) alternative forensic marker systems; (ii) forensic applications of supplementary non-core STRs; (iii) population genetics and population databases; (iv) massively parallel sequencing and emerging technologies; (v) validation and implementation challenges; and (vi) ethical and legal considerations.

## 2. Overview of the Main Genetic Marker Classes

Forensic genetics has progressed through successive phases: exploration (1985–1995), standardization (1995–2005), growth (2005–2015), and sophistication (2015 onwards), expanding its capacity to address diverse biological samples and investigative questions [5]. Although autosomal short tandem repeats (A-STRs) remain the gold standard for routine human identification, no single marker system is optimal for all forensic applications. Marker selection therefore depends on the case context, the investigative question, and the quantity and integrity of the available DNA [7,22]. Table 1 summarizes the principal forensic genetic markers, including their amplicon size, mutation rate, discriminatory power, main applications, and limitations.

### 2.1. Autosomal Short Tandem Repeats (A-STRs)

Autosomal short tandem repeats (A-STRs) are biparentally inherited, multiallelic markers located on the autosomes and composed of tandemly repeated motifs of 2–7 base pairs [9]. Their abundance, high polymorphism, sensitivity, and compatibility with established analytical workflows make them the gold standard for human identification [7]. However, their relatively long amplicons and susceptibility to stutter artifacts can complicate the analysis of degraded, low-template, or mixed samples, and standard A-STR panels may provide insufficient statistical power in complex mixtures or distant kinship cases [6,7,15]. Their biological basis, forensic applications, and technical limitations are discussed further in Section 3.

### 2.2. Sex Chromosome Markers: Y-STRs and X-STRs

Sex chromosome markers provide lineage-specific information that complements autosomal STR analysis.

Y-chromosomal STRs (Y-STRs) are male-specific markers inherited as linked haplotypes through the paternal lineage [23,35]. They are particularly useful for analyzing male–female mixtures, paternal kinship, missing-person cases, and disaster victim identification. Because Y-STRs generally do not recombine, match probabilities are estimated using haplotype frequencies rather than the product rule [23]. Their main limitation is the reduced ability to distinguish males from the same paternal lineage, although rapidly mutating Y-STRs improve discrimination between closely related males [22,24].

X-chromosomal STRs (X-STRs) exploit the distinct inheritance patterns of the X chromosome and are particularly informative in complex or deficient kinship cases involving females, or for identification purposes when reference parental data are absent [26,36]. However, their interpretation requires consideration of linkage, recombination, and haplotype frequencies, and they are uninformative for father–son relationships [24,37].

### 2.3. Mitochondrial DNA (mtDNA)

Mitochondrial DNA (mtDNA) is a 16,569 bp circular, extranuclear genome present in high copy numbers within cells, which increases its persistence in highly degraded samples [38]. It is therefore particularly useful for analyzing skeletal remains, charred bones, teeth, rootless hair shafts, missing persons, and disaster victim identification cases in which nuclear DNA is compromised [7,15].

Because mtDNA is maternally inherited, remains largely unchanged and is shared among maternal relatives, it is primarily used for lineage assessment rather than individualization. Its interpretation may be complicated by heteroplasmy, a phenomenon in which more than one mtDNA type is present within an individual’s cells [39,40].

### 2.4. Biallelic Markers: Single-Nucleotide Polymorphisms (SNPs) and Insertion/Deletion (InDel) Polymorphisms

Single-nucleotide polymorphisms (SNPs) are predominantly biallelic variants distributed across coding and non-coding regions of DNA [28,38]. According to their forensic application, they may be classified as identity-, lineage-, ancestry-, or phenotype-informative markers [1]. Their short amplicon size and low mutation rate make them suitable for degraded DNA analysis, lineage tracing, and multigenerational kinship investigations [28,38]. Insertion/deletion polymorphisms (InDels) are biallelic markers defined by the insertion or deletion of one or more nucleotides [29]. Their low mutation rates and short amplicons make them useful for degraded samples, kinship analysis, and population studies, while their length-based variation allows analysis using methods compatible with STR typing [15,41].

It is important to note that because SNPs and InDels are predominantly biallelic, each locus provides only a limited number of genotype combinations, increasing allele sharing among contributors in mixed samples. This reduces their ability to distinguish contributor-specific profiles compared with multiallelic markers such as STRs and microhaplotypes, which offer greater heterozygosity and a larger number of possible allele combinations [32,42]. Consequently, larger SNP and InDel panels are generally required to achieve adequate discriminatory power [28,43].

### 2.5. Compound Markers: Microhaplotypes and Multi-InDels

Microhaplotypes (MHs) are short compound markers comprising two or more tightly linked SNPs analyzed as a single multiallelic haplotype [31]. Typically, shorter than 300 bp, they combine the low mutation rate and short amplicon size of SNPs with the higher heterozygosity and multiallelic structure of STRs [32]. Their absence of stutter artifacts and suitability for multiplexing make them useful for mixture deconvolution, individualization, ancestry inference, and kinship analysis [44]. Their main limitation is their reliance on massively parallel sequencing and the associated implementation requirements [32,33].

Additional compound markers include SNPs closely linked with STRs (SNP-STR), InDels in proximity to STRs (DIP-STR), InDels associated with SNPs (DIP-SNPs), and several InDels that are tightly linked together (Multi-InDels), which combine closely linked variants to increase polymorphism and forensic informativeness [31,34].

Collectively, these marker systems illustrate the breadth of the forensic genetic toolkit and the trade-offs inherent to each marker class. Although each addresses specific forensic scenarios, none replaces the established role of STRs in routine casework. Their limitations instead reinforce the centrality of STR-based workflows and the practical need to extend them. In this context, supplementary non-core STRs represent a pragmatic solution, increasing the discriminatory potential of standard forensic panels while maintaining compatibility with existing STR infrastructure, methodologies, and databases. The following sections therefore focus on their biological characteristics, forensic applications, population genetics, and implementation requirements.

## 3. Short Tandem Repeats: The Gold Standard

STRs, also known as microsatellites, are short DNA sequences repeated multiple times in a head-to-tail configuration [45]. These sequences can be repeated in tandem up to 100 times within the genome [17]. STRs constitute approximately 3% of the total human genome, with an average occurrence of one STR per 2000 bp [9]. The majority of STRs are in gene deserts or non-coding regions. Shi et al. (2023) [46] found that over 90% are located in intronic (58.5%) and intergenic (34.5%) regions. Since most STRs are located in the non-coding regions of the human genome, these sequence variations do not affect an individual’s health or phenotype, allowing the mutations to be safely inherited and accumulate significant diversity within populations [47].

STRs can be classified based on their repeat units as di-, tri-, tetra-, or pentanucleotide repeats. Although dinucleotide repeats are the most prevalent STR class in the human genome, forensic science preferentially utilizes tetranucleotide and pentanucleotide repeat motifs because of their abundance and stability, which results in a significantly lower likelihood of polymerase slippage and reduced stutter artifact generation [17]. STRs are variable in the length of their repeating units, the number of repetitions, and their regular incremental repeat patterns. Based on these patterns, STRs can be categorized as simple repeats, which consist of units with identical length and sequence; compound repeats, which comprise two or more adjacent simple repeats; or complex repeats, which encompass multiple repeat blocks with varying unit lengths and sequences [12]. This structural diversity influences both the allelic range and the analytical behavior of individual loci within forensic workflows.

Autosomal STRs are inherited biparentally, meaning that an individual receives one allele from each parent, resulting in co-dominant expression, where the individual is either homozygous or heterozygous at a given locus [13]. STRs exhibit mutation rates 10,000 to 100,000 times higher than the average across the genome, with mutation rates ranging from 10^−6^ to 10^−2^ per generation [38,45]. The fundamental premise of STR variation is that individuals differ in the exact number of repeating units they possess at a given locus, as STRs are fundamentally polymorphic in their length, but as advanced sequencing has revealed, also in their internal sequence [17,47]. Multiple potential mechanisms have been proposed to account for the high mutation rate of STRs, with polymerase template slippage being the primary mutational mechanism responsible for alterations in STR length [38]. During the process of DNA replication, the template strand and the newly synthesized strand may temporarily dissociate and misalign, resulting in the insertion or deletion of one or more repeat units in the allele, which is subsequently inherited by the offspring. Changes involving a single repeat unit are more prevalent than those involving multiple repeats. Over generations, these changes lead to a diverse range of allele lengths. These findings underpin the currently recognized mutational framework, known as the Stepwise Mutation Model [37,38]. Sister chromatid exchange, along with point mutations, can also contribute to the expansion, contraction, or fragmentation of repeat segments, albeit to a lesser extent [37,38]. Research indicates that the frequency of these mutational events increases with allele length. Longer alleles that remain uninterrupted are particularly unstable and frequently mutate into shorter forms, whereas alleles of intermediate size have an equal probability of either expanding or contracting [37,48].

STRs are the workhorses of forensic genetics. As reviewed by Gutiérrez-Hurtado et al. (2025) [1], the key features of STRs are (I) their presence in non-coding regions of the genome, (II) their abundance across both autosomes and sex chromosomes, (III) their highly polymorphic DNA sequences, with polymorphism rates exceeding 70%, (IV) their capacity to vary in size among individuals, providing a discrimination power greater than 0.8 without affecting health, and (V) the technical simplicity of their rapid analysis through PCR-based technology and CE with automated fluorescent detection. Owing to these attributes, the now-defunct Forensic Science Service embarked on a comprehensive search for novel STR candidates, which ultimately led to their standardization in forensic practices.

### 3.1. Standardization: CODIS, the European Standard Set

To fully leverage the potential of STRs, the forensic community recognized the need for rigorous standardization, which served as the primary impetus for establishing core STR sets. This initiative was led by collaborative efforts from organizations such as the European DNA Profiling Group (EDNAP), European Network of Forensic Science Institutes (ENFSI), and Scientific Working Group on DNA Analysis Methods (SWGDAM). Employing a standardized set of loci ensures reproducibility and equitable justice outcomes and guarantees that DNA profiles are compatible across jurisdictions. This compatibility facilitates efficient data management, enables cross-border sharing of criminal intelligence, and allows automated searching within extensive national databases [12,13]. In April 1995, the UK launched the first national DNA database, based on SGM, a second-generation multiplex that included six STR loci and the sex-determining amelogenin [49]. This initiative set the stage for the European Standard Set (ESS), which initially featured four loci and quickly expanded to seven core loci by 1999, namely TH01, vWA, FGA, D21S11, D3S1358, D8S1179, and D18S51 [11]. Concurrently, the United States embarked on a significant standardization effort, with the FBI announcing in 1998 the selection of 13 core STR loci to form the basis for the Combined DNA Index System (CODIS) and the National DNA Index System (NDIS), which included CSF1PO, FGA, TH01, TPOX, vWA, D3S1358, D5S818, D7S820, D8S1179, D13S317, D16S539, D18S51, and D21S11 [9].

The rapid success of these databases necessitated the inclusion of additional loci to reduce the statistical likelihood of coincidental matches, facilitate more complex investigations of missing persons, and enhance compatibility for international data sharing. The subsequent expansion of the ESS loci was prompted by the Prüm Treaty of 2005, an agreement signed by several European member states to enhance cross-border collaboration through information exchange [11]. The ESS was upgraded to 12 loci in 2010 [10], followed by the United States, which expanded its core loci to 20 in 2017 [12].

In general, the selected loci were derived from non-coding intronic and intergenic regions, ensuring that forensic profiling does not inadvertently disclose sensitive medical information or phenotypic traits in compliance with ethical and legal mandates [3,4]. The selection was also governed by stringent scientific criteria that represent desirable STR characteristics. Firstly, selected loci are widely distributed across the human genome and are chosen for high polymorphism to maximize discriminatory power, typically exhibiting four-nucleotide motifs and at least 16 different alleles [50]. Secondly, markers are required to segregate independently, being located on different chromosomes or sufficiently distant on the same chromosome to ensure linkage equilibrium, thereby allowing the mathematical combination of individual locus frequencies using the product rule [12]. Thirdly, loci must demonstrate robust compatibility with multiplex PCR and CE workflows for practical implementation across forensic laboratories [9]. These characteristics should be considered when designing new STR sets.

Currently, commercial multiplex kits incorporate markers from both expanded CODIS and ESS sets. These kits can simultaneously co-amplify overlapping markers from both sets, thereby proving highly effective in resolving routine forensic cases and establishing a universal standard for forensic data exchange [12].

### 3.2. Analytical Methodology

Forensic STR typing, based on multiplex PCR followed by CE, is the standard procedure in forensic sciences. The conventional DNA typing process involves a rigorous, sequential methodology: sample collection, DNA extraction, quantification, multiplex PCR amplification, CE separation, and data analysis [8]. During amplification, specialized fluorophore-labeled primers simultaneously amplify multiple STR loci in a process known as multiplexing [51]. The amplified products are then subjected to CE, in which an applied electric field drives the fragments through a polymer-filled column, separating them by size. The fluorophore labels are subsequently excited by a laser, prompting them to emit signals that appear as peaks on an electropherogram. This data is analyzed using software that compares it to a standardized allelic ladder and assigns each peak to a specific allele designation based on the number of repeat units [17,38].

Despite the availability of advanced technologies, the interpretation of STR profiles from highly degraded, low-quantity DNA or mixed contributors remains challenging. In samples with low-template DNA, larger amplicons often fail to amplify, and stochastic effects during PCR can result in heterozygote imbalance, allele drop-out (where existing alleles are not observed), or drop-in (where non-existing alleles are observed) [13]. Furthermore, the PCR process inevitably produces stutter artifacts, which are minor peaks typically one repeat unit shorter than the actual template strand, caused by slipped-strand mispairing. These artifacts significantly complicate the interpretation of mixed DNA profiles, particularly when their peak heights are similar to those of the true alleles from minor contributors [17]. Although CE is currently the standard method, alternative techniques such as MPS, which address many of these challenges, are expected to shape the future of STR analysis, as outlined in Section 6 [52].

## 4. Non-Core and Supplementary STR Loci

Several commercial multiplex systems are used internationally, including the GlobalFiler^TM^ kit (Applied Biosystems, Foster City, CA, USA), the PowerPlex^®^ Fusion system (Promega, Madison, WI, USA), and the Investigator^®^ 24plex QS kit (QIAGEN, Hilden, Germany), each targeting 22–27 loci and encompassing the full CODIS and ESS core sets. For complete standard profiles generated by these systems, random match probabilities routinely fall below one in a billion, forming the statistical foundation of forensic DNA evidence worldwide [5,34]. While these systems provide essential genetic data for routine casework with considerable discriminatory power, they exhibit defined limitations in forensically complex scenarios, such as interpreting complex DNA mixture profiles and analyzing distant kinship [15,16,17].

This underscores the need for additional markers, such as supplementary sets of STR markers referred to as non-core STRs, to augment the data provided by the core loci set. This enables the extraction of multidimensional information from a single STR profile, thereby aiding in the resolution of complex forensic challenges [20]. Researchers and commercial developers have characterized new supplementary non-core STR loci throughout the human genome, and their incorporation in practice substantially increases combined discriminatory power, provides the statistical weight necessary to resolve complex pedigrees, and contributes information relevant to population structure [20].

The following sections examine in detail how these supplementary non-core STRs are applied to the most challenging forensic scenarios, how their population genetics are characterized, and what validation and standardization frameworks govern their implementation.

### 4.1. Forensic Applications of Supplementary STR Panels

#### 4.1.1. Complex Kinship Resolution and Deficiency Cases

One of the primary applications is resolving complex pedigree disputes. The proportion of alleles shared identically by descent naturally decreases with each degree of relationship. In distant kinship analyses, such as those involving half-sibling, grandparent-grandchild, or avuncular relationships (uncle/aunt–nephew/niece), the calculated likelihood ratio frequently falls within an inconclusive range due to substantial overlap in the likelihood ratio distributions of true relatives and unrelated individuals [53]. It is also important to consider the impact of natural mutation events, which can significantly reduce the Paternity Index and complicate the determination of whether the mismatch is due to a natural mutation or evidence of non-paternity [54].

Numerous studies indicate that the incorporation of highly polymorphic non-core STRs offers the additional statistical robustness required to address this uncertainty. Huang et al. (2024) [20] evaluated a 108-STR panel (comprising 20 CODIS and 88 non-CODIS STRs) using whole-genome sequencing and found that the inclusion of non-CODIS STRs significantly diminished the overlap in Identical by State scores among distant relatives up to the third generation, achieving a perfect accuracy rate of 100% in distinguishing parent-offspring and full-sibling relationships. A separate study, which examined numerous intricate simulated pedigree scenarios involving trios with close familial connections, demonstrated that standard panels are susceptible to false inclusions, particularly when the biological parents are consanguineous or when the alleged parent is a close relative. The combined use of 20 CODIS STRs and 21 non-CODIS STRs showed sufficient Cumulative Paternity Index power to effectively resolve paternity testing, even in these complex incestuous mating cases [18].

Supplementary STRs can also play a crucial role in resolving paternity cases where a biological parent is unavailable. In this context, Zhang et al. (2020) [55] introduced the SiFaSTR 21plex_NCII typing system, which incorporates 18 novel non-CODIS STR loci specifically designed to meet the high demand for locating lost siblings and children in China, due to historical reasons. The combined exclusion power in duos and trios exceeded 0.9997347 and 0.9999997, respectively, demonstrating both reliability and accuracy, and providing sufficient statistical power for complex parentage testing where traditional commercial kits are inadequate. Similarly, various authors have independently evaluated different non-CODIS commercial kits in Asian populations, where there is a high prevalence of complex parentage cases involving missing children or distant relatives. Panels such as the Microreader 23HS Plex ID System [56], the SureID 23comp [57], the Goldeneye^TM^ DNA ID 22NC kit [58], and others [54], which mainly incorporate non-CODIS STRs, can achieve combined exclusion probabilities exceeding 0.9999999, making them robust supplementary tools.

#### 4.1.2. Analysis of Degraded DNA and Disaster Victim Identification

Forensic samples obtained from challenging environments or mass disaster scenarios often exhibit significant DNA degradation due to hydrolytic, oxidative, enzymatic, or microbial processes [14,15,17]. Standard STR profiling is particularly susceptible to degradation because it depends on amplicons ranging from approximately 100 to 400 base pairs. As DNA degrades, larger amplicons are less likely to amplify, resulting in locus dropout and incomplete profiles [14,15].

Research has demonstrated that panels incorporating non-core STRs can offer advantages. The STRtyper-32G kit, which comprises 20 CODIS loci and 10 additional loci, has been effective in generating complete DNA profiles from 50 aged bloodstains, ranging from 4 to 8 years old [59]. In a study by Adnan et al. (2025) [60], the AGCU EX-38 typing system, which includes 38 loci with 9 STRs within 200 base pairs and 14 STRs within 300 base pairs, was evaluated. This size range enhances performance in cases involving degraded samples, as the system successfully produced complete profiles from severely degraded formalin-fixed and paraffin-embedded biopsies and from all samples aged 10 years, despite reduced peak heights with larger fragment sizes [60]. Comparable outcomes were achieved using the SiFaSTR^TM^ 23-plex system, which integrates 21 autosomal STRs, as it also fully genotyped severely degraded samples and all 10-year-old samples [61].

Most notably, the literature highlights the importance of mini-STRs, with amplicons ranging from 50 to 150 bp, in improving the recovery of genetic information from degraded DNA. These shorter target sequences are statistically more likely to remain intact in fragmented samples [9]. Burguete-Argueta et al. (2016) [62] examined six non-CODIS mini-STRs and SE33 in Mexican Mestizo and Amerindian populations. Owing to their smaller PCR amplicons, these markers successfully enhanced analysis of degraded DNA samples, where standard CODIS markers yielded partial or failed profiles, while also showing a high combined power of discrimination and a high mean power of exclusion.

Coupling mini-STRs with MPS techniques facilitates the design of overlapping, extremely short amplicons for all loci simultaneously, significantly enhancing allele recovery [52]. Research using multiplex systems such as the maSTR assay [59] and the NHID Mini25A [63] has demonstrated their ability to genotype ≥ 80% of targeted loci, with enhanced performance compared to conventional methodologies. Numerous STRs are also being incorporated into common commercial kits, such as the ForenSeq^TM^ DNA Signature Prep and the Precision ID GlobalFiler^TM^ NGS STR Panel [64].

The incorporation of various marker types, such as non-core STRs, into a single multiplex system offers a beneficial strategy for examining degraded samples. Lu et al. (2026) [65] validated a 436-plex MPS system with 88 STRs and 348 SNPs. This panel demonstrated remarkable robustness in simulated degradation tests using DNase I, achieving genotyping accuracy rates of 99.42% for STRs and 100% for SNPs. Sensitivity evaluations revealed that complete profiles could be obtained at concentrations as low as 0.125 ng for STRs and 0.0625 ng for SNPs. These findings suggest that STR profiling and SNP genotyping should be regarded as complementary rather than competing methodologies.

In contexts such as Disaster Victim Identification, mass fatality incidents, and the analysis of mass graves, where remains are often extensively degraded, mixed, or fragmented, such as ancient skeletal remains, traditional DNA profiling frequently encounters significant challenges [15]. Reference samples are often unavailable, and identifications must rely on distant surviving relatives [66,67]. The combination of short-amplicon supplementary STRs provides both the environmental robustness and the immense statistical power necessary to secure accurate kinship matches.

#### 4.1.3. Complex DNA Mixture Deconvolution

The interpretation of mixed biological samples containing DNA from multiple contributors remains a significant challenge in forensic genetics. Standard CE-based methods often encounter difficulties with unbalanced mixtures, including allele drop-out, allele drop-in, allele masking between contributors, and stutter peaks that can obscure the minor contributor’s profile [17,22,32]. Expanding the testing panel to include numerous highly polymorphic non-core STRs mathematically increases the probability of identifying unique, unshared alleles for the minor contributor [20,68].

Novroski et al. (2019) [68] explored a 73-plex of highly polymorphic STRs and a 20-plex subset (20Plex). Through in silico evaluation of two-person mixtures, the authors found that the non-core 20Plex significantly outperformed the 20 CODIS core loci in resolving component contributors, yielding an 82.6% increase in the proportion of loci with four fully resolved alleles when using length-based data.

Another study assessed the deconvolution capabilities of an expanded panel comprising 20 CODIS and 88 non-CODIS STRs on complex multi-contributor mixtures involving 2 to 8 individuals. The panel demonstrated perfect accuracy in predicting the Number of Contributors for mixtures of 2 and 3 individuals and accurately predicted it for mixtures of up to 4 individuals. When evaluated under probabilistic genotyping frameworks for highly complex mixtures with more than six contributors, the inclusion of the 88 non-CODIS STRs significantly enhanced evidential strength, resulting in likelihood ratio values exceeding 1,000,000. In both evaluations, the standard CODIS core set produced substantial errors [20].

The incorporation of additional supplementary markers seeks to further enhance the analysis. Fan et al. (2021) [69], Liu et al. (2024) [70], and Lu et al. (2026) [65] developed comprehensive multiplex panels (133-Plex, 193-plex, and 436-plex, respectively) that incorporate non-core STRs alongside CODIS STRs, X/Y-STRs, or SNPs to exploit multidimensional genetic information. These expanded panels exhibited robust detection capabilities for minor contributors in male–male, male–female, and female–female mixtures at different ratios. The inclusion of sex-specific and supplementary non-core markers enabled the detection of scarce minor contributors even in extreme 1:99 male–female mixtures.

It is noteworthy that, in the context of mixtures, sequence-based analysis offers an important additional dimension of data. This sequence-level resolution enables analysts to successfully deconvolute mixtures and accurately differentiate true minor contributor alleles from the stutter artifacts of the major contributor, thereby significantly enhancing mixture deconvolution capabilities [71,72]. Furthermore, the statistical interpretation of these complex profiles is conducted using probabilistic genotyping software, which models all potential genotype combinations for each contributor and calculates likelihood ratios accordingly. Commercially validated platforms, including STRmix, TrueAllele, and EuroForMix, can incorporate extended non-core STR loci, provided that population allele frequency data and locus-specific stutter parameters have been empirically established for the target population [22].

### 4.2. Global Population Genetics and Biogeographical Ancestry Inference of Non-Core STR Loci

Before introducing any additional markers into forensic casework, forensic geneticists must develop allele frequency databases specific to the population under study. This step is vital for accurately calculating match probabilities, underscoring the significance of population genetics [73]. Extensive research has examined supplementary STR markers across various geographic populations, revealing distinct patterns of genetic diversity and population structure. These studies also assessed their forensic applicability in different human populations, showing that these loci exhibit high levels of polymorphism and genetic diversity within a globally diverse demographic [64].

Huang et al. (2024) [20] offer an extensive overview with a catalog of 178 non-CODIS STRs derived from whole-genome sequencing data across global populations, demonstrating that these have polymorphism information content, observed heterozygosity, and match probabilities on par with CODIS STRs. Using a Random Forest machine learning model to infer biogeographical ancestry, the panel significantly outperformed the standard CODIS loci, achieving accurate ancestry classification for African, East Asian, and South Asian samples. Notably, 18 of the 20 most informative loci were non-CODIS markers. Population structure analyses reflected expected patterns, including higher STR diversity in African populations, the lowest diversity in Oceanian populations, and widespread admixture in American populations, which directly mirrors the progressive loss of genetic variability associated with historical out-of-Africa bottlenecks [50]. A separate study demonstrated that employing a combination of the PowerPlex^®^ Fusion System and non-core QIAGEN Investigator^®^ HDplex kits within a 32 STR panel effectively differentiated and categorized individuals across five super-populations in the Human Genome Diversity Project—Centre d’Étude du Polymorphisme Humain (HGDP-CEPH) panel [74].

Several studies have been conducted on European populations. Červenák et al. (2021) [75] evaluated nine non-CODIS STR markers from the Investigator^®^ HDplex kit within the Slovak population, demonstrating that these markers alone are sufficient to reliably cluster different European populations and distinctly separate them from American, Asian, and African continental clusters using Multidimensional Scaling and Neighbor-Joining phylogenetic trees. Other studies employing the same kit, such as those by Turrina et al. (2015) [76], Wojtkiewicz et al. (2016) [77], and Zieger et al. (2021) [78], in North Italian, Polish, and Swiss populations, respectively, all reported high combined powers of discrimination and exclusion. Another set of 15 extended non-core loci was validated for an Austrian Caucasian sample, achieving a combined probability of exclusion of 0.99999998 without significant linkage to standard loci [79]. Supplementary STRs can also distinguish between very closely related populations, as evidenced by a study comparing 44 autosomal STR loci between English and Irish populations, which found a very close genetic relationship that supports merging their allele frequency databases [80].

Research on regional populations substantiates the forensic application of non-core STR markers, which are frequently employed to elucidate the intricate population structure of China and its neighboring regions. Panels of non-core STRs analyzed in the Han Chinese, the largest ethnic group in China [58,81,82] as well as in ethnic minorities such as the Central Asian-clustering Kyrgyz [54], Tibetan and Yi minority ethnic groups [83] and the small, endogamous Baoan group [84] reveal the phylogenetic distinctions among communities within the same country and underscore the necessity for population-specific databases to ensure accurate forensic identification in demographically unique communities. On a broader East Asian scale, Japanese and Korean populations show a close genetic relationship across Investigator^®^ HDplex loci, with notable differentiation only when compared with geographically distant populations such as Argentines and Northern Italians [85,86].

Research focusing on other population subsets is less prevalent. Dash et al. (2021) [87] investigated the efficacy of highly polymorphic non-CODIS markers within the Central Indian population, revealing significant heterozygosity (>0.80). Utilizing a Neighbor-Joining tree, they demonstrated that the Central Indian population constitutes a distinct branch, entirely separate from East and Southeast Asian populations. A study in Mexican Mestizo and Amerindian populations using 7 non-CODIS STRs confirmed that these markers provide high combined power of discrimination and, through Principal Coordinates Analysis, elucidated the complex genetic structure of Mestizos, positioning them genetically between Amerindian and Spanish ancestors [62].

Despite the substantial evidence available, significant geographic gaps remain in population data, particularly concerning African and Middle Eastern groups, which are notably underrepresented in published non-core STR datasets. This limitation is especially problematic because these populations often exhibit considerable genetic diversity and population substructure that may not be adequately represented by European or East Asian reference databases. Consequently, the use of inappropriate allele frequency estimates may lead to inaccurate match probabilities and likelihood ratio calculations, potentially affecting the statistical weight and reliability of forensic evidence. Expanding population-specific databases for these underrepresented groups should therefore be regarded as a prerequisite for the broader forensic implementation of supplementary STR panels rather than merely a future research objective [71,88,89].

Several factors likely contribute to the persistent underrepresentation of African and Middle Eastern populations in non-core STR databases, including limited forensic genetic infrastructure, unequal research funding distribution, reduced access to high-throughput genotyping technologies and the historical concentration of forensic population studies in Europe, North America and East Asia [90]. Addressing these disparities will require coordinated international efforts aimed at supporting population sampling initiatives, developing regional reference databases and promoting collaborative research networks involving currently underrepresented regions.

It is crucial to recognize that employing non-core STRs for geographic tracing differs from Forensic DNA Phenotyping, which utilizes aiSNPs for characteristic and biogeographical inference and follows a distinct analytical method. STRs offer insights through length polymorphism diversity rather than ancestry-informative variants. The forensic application of genetic ancestry inference raises significant ethical concerns, as described in Section 9, and any use of non-core STRs in these contexts must adhere to ethical and regulatory standards [42].

## 5. Case Study: Investigator^®^ HDplex Kit as a Model for Supplementary Non-Core STR Implementation

An exemplary and well-documented instance of incorporating supplementary non-core STRs in forensic applications is the Investigator^®^ HDplex kit developed by QIAGEN, Hilden, Germany. This multiplex system is specifically designed to facilitate complex human identification and kinship testing by simultaneously amplifying 12 autosomal STRs and the sex-determining Amelogenin in a single multiplex PCR assay, most of which are not included in commonly used commercial panels [53]. It includes two standard core markers (D12S391, D18S51) and ten highly polymorphic supplementary loci not present in either CODIS or ESS, namely SE33, D2S1360, D3S1744, D4S2366, D5S2500, D6S474, D7S1517, D8S1132, D21S2055, and D10S2325 [75]. The integration of established core markers with supplementary loci serves dual purposes, as it enables direct compatibility of Investigator^®^ HDplex results with current forensic database profiles, while the non-core loci provide additional discriminatory power necessary in cases where standard panels are insufficient [91]. Table 2 displays the Investigator^®^ HDplex loci, including their core set status, their chromosomal positions, repeat motifs, and locus-specific artifacts as reported in the literature.

### 5.1. Global Population Validation and Forensic Performance

Numerous studies utilize the Investigator^®^ HDplex kit. In a comprehensive study employing this kit, the researchers genotyped 941 individuals from 51 populations, encompassing seven continental groups within the HGDP-CEPH panel. These loci demonstrated significant informativeness globally, with at least six loci exhibiting remarkable forensic discrimination capabilities in each continental population group [67].

To avoid a purely descriptive enumeration of individual studies, the main population-based evaluations of the Investigator^®^ HDplex kit are summarized in Table 3.

Europe is particularly well represented in Investigator^®^ HDplex validation studies, including Dutch, Italian, Polish, Swiss and Portuguese populations [75,76,77,78,98,99]. Across these studies, the loci consistently exhibited high levels of polymorphism, combined power of discrimination and power of exclusion, while generally conforming to Hardy–Weinberg equilibrium expectations. These findings support the robustness of the kit as a supplementary tool for human identification and kinship testing in European populations.

Similar findings have been reported in East Asian populations, including South Korean, Japanese and Han Chinese cohorts, where the Investigator^®^ HDplex kit consistently exhibited high forensic informativeness and enhanced discriminatory capacity when combined with standard STR panels [81,82,85,86].

Studies conducted in geographically diverse populations outside Europe and East Asia, including Argentine, Ecuadorian and Russian cohorts, have likewise demonstrated high levels of polymorphism, combined power of discrimination and forensic informativeness [91,100,101]. Particularly noteworthy is the Argentine study, which not only confirmed the forensic utility of the Investigator^®^ HDplex kit but also established reference allele frequency databases and identified regional genetic patterns relevant for forensic applications [91].

Collectively, these studies demonstrate that the Investigator^®^ HDplex kit exhibits consistently high forensic performance across diverse populations worldwide. However, important gaps remain for African and Middle Eastern populations, highlighting the need for additional population-specific databases before broader forensic implementation.

As outlined in Section 4, non-core panels are highly valuable in forensic analysis, especially when standard core STR multiplexes produce ambiguous results, and the Investigator^®^ HDplex kit is no exception to this. Numerous studies highlight the kit as a highly effective supplementary tool, especially for investigating distant familial relationships when key individuals are unavailable or when genetic inconsistencies arise due to mutations. Turrina et al. (2016) [53] assessed whether the adoption of STR combinations (Identifiler+Investigator^®^ HDplex and Fusion+Investigator^®^ HDplex) positively influenced the resolution of kinship relationships in 300 pairs of related and unrelated individuals. Expanding from 15 to 24 loci enhanced the distinction between relatives and non-relatives, particularly for full siblings, and reduced the overlap between the false-negative and false-positive curves, known as the grey zone. In another study by the same author, mutation rates were estimated in 84 confirmed family trios at 2.94 × 10^−3^ per locus per generation, and the 12 loci proved highly informative, with a combined power of discrimination of 0.999998 [76]. Tillmar et al. (2014) [102] examined the efficiency of the Investigator^®^ HDplex kit against supplementary biallelic markers, such as SNPs and insertion/deletion polymorphisms, and concluded that the Investigator^®^ HDplex kit was the most efficient set of supplementary markers not only for standard paternity investigations but also for highly complex scenarios, where a close biological relative is suspected to be the alternative father, including cases involving natural mutations to confirm true paternity. Overall, the studies underscore the significant utility of the Investigator^®^ HDplex kit as an auxiliary tool, particularly in complex kinship analyses.

### 5.2. Technical Artifacts and Analytical Considerations

While the Investigator^®^ HDplex kit is globally recognized for its discriminatory capabilities, its widespread use has revealed several technical artifacts specific to certain loci that forensic analysts must consider when interpreting profiles. These artifacts do not necessarily compromise the kit’s effectiveness but necessitate that laboratories specifically characterize them and develop informed interpretation guidelines [97]. Generally, because it uses an older four-dye chemistry, the kit employs amplicons ranging from 70 to 475 bp, which can render it susceptible to DNA degradation [98].

At the locus level, interpretative anomalies frequently complicate analysis. For example, at the D10S2325 locus, Westen et al. (2012) [98] reported an approximate 0.5 nucleotide peak shift relative to the allelic ladder, resulting in allele binning failures. Other independent studies have identified that large off-ladder alleles and plus-stutter peaks at D10S2325 can migrate into the size range of the adjacent SE33 locus, potentially leading to misinterpretation as triallelic patterns or off-ladder SE33 alleles, which could cause significant genotyping errors [81,82,85].

Peak height imbalance is another common phenomenon in STR analysis and refers to substantial differences in signal intensity between the two alleles of a heterozygous genotype. In general, this imbalance results from unequal amplification efficiency during PCR, particularly when the two alleles differ considerably in fragment length. Shorter alleles tend to amplify more efficiently than longer alleles, leading to reduced peak height ratios [103,104]. The effect may be further exacerbated in low-template or degraded DNA samples, where stochastic amplification effects can increase allelic imbalance and, in extreme cases, contribute to allele dropout [11,105]. For example, the D21S2055 locus has been demonstrated to exhibit severe peak height imbalance, with peak height ratios often falling below 60% in heterozygous genotypes due to differential amplification efficiency across alleles with large size differences, as documented by Tillmar et al. (2013) [96], Zieger et al. (2021) [78], and Lin et al. (2026) [97], the latter reporting an extreme off-ladder allele (57.2). This imbalance may restrict the utility of D21S2055 for mixture and trace analysis. At D2S1360, Inokuchi et al. (2017) [94] identified a common G > A transition at the primer binding site of the D2S1360 locus in the Japanese population, causing severe heterozygote peak imbalance and potential complete allele dropout, resulting in apparent homozygosity and risking false exclusions in kinship testing or complicating mixture interpretation. Analysts are advised to suspect this variant when the peak height ratio falls below 0.45. Notably, the prevalence of this variant in other populations is a gap relevant to laboratories implementing the kit.

To address these challenges, laboratories must implement specific practical strategies and adjust their statistical parameters before applying them in actual casework. For instance, Westen et al. (2012) [98] highlighted that the default minus-one-repeat stutter-ratio filters provided by manufacturers are often set too low. This can lead to the erroneous identification of artificial stutter peaks as true alleles, even in high-quality DNA samples. They recommend that laboratories establish empirical stutter thresholds internally using samples that reflect the population, rather than relying on the manufacturer’s default settings. To address other locus artifacts, the authors generally advised conducting in-house reaction optimizations and considering PCR confirmation or cross-verification with alternative kits for problematic loci [98].

Furthermore, because Investigator^®^ HDplex is frequently used as a supplementary tool alongside other multiplexes, the physical co-localization of loci must be considered. Several Investigator^®^ HDplex markers are located on the same chromosomes as standard core STRs, creating syntenic locus pairs, the most relevant of which are summarized in Table 4.

Despite this physical proximity, independent evaluations have consistently demonstrated an absence of significant allelic association or linkage disequilibrium at the population level, allowing for safe application of the product rule when calculating combined random match probabilities for unrelated individuals in routine casework [91,98]. However, physical linkage becomes a critical consideration in complex kinship and familial testing. In these contexts, recombination fractions and genetic distances should be incorporated into statistical calculations, since ignoring the genetic coupling of closely situated loci, such as vWA and D12S391 [106], may lead to substantial overestimation of likelihood ratios by up to 100-fold in pedigree and sibship analyses, as documented by Tillmar et al. (2013) [96] and Westen et al. (2012) [98].

Overall, the technical artifacts identified in multiple independent validation studies of the Investigator^®^ HDplex kit underscore the inadequacy of relying solely on manufacturer specifications during implementation. Laboratories adopting the kit must conduct comprehensive in-house validation, which, although demanding, is crucial for the responsible and legally defensible use of the kit in operational casework.

## 6. The Synergy of Non-Core STRs and Massively Parallel Sequencing

The analytical capabilities of non-core STRs are significantly enhanced when integrated with MPS [20]. MPS identifies length- and sequence-based polymorphisms and addresses CE’s inherent limitations, such as its restricted multiplexing capacity of approximately 20 to 30 markers due to constraints on fluorescent dye channels and the need for non-overlapping amplicon size ranges [52]. It enables the simultaneous multiplexing of hundreds of markers in a single assay, such as STRs, SNPs, InDels, microhaplotypes, and even mitochondrial genome data, while facilitating the acquisition of their full sequences. For example, the MGIEasy Signature Identification Library Prep kit comprises 52 autosomal STRs, 27 X-chromosomal STRs, 48 Y-chromosomal STRs, 145 iiSNPs, 53 aiSNPs, 29 piSNPs, and the hypervariable regions of mtDNA [107]. By utilizing shorter amplicons, it significantly enhances the analysis of highly degraded or compromised DNA, thereby optimizing the retrieval of genetic data [15]. Critically, MPS surpasses CE by analyzing STRs at the level of precise nucleotide sequences, revealing isoallelic variation within alleles of the same length [52]. This also addresses homoplasy, where fragments of similar size but different sequence composition produce a single CE peak and are incorrectly interpreted as homozygous [108]. By capturing sequence-level variation within both the core repeat motif and flanking regions, MPS substantially increases the effective allele count and observed heterozygosity of non-core loci, directly translating into greater discriminatory power [109].

Numerous studies report a substantial rise in allelic diversity when shifting to sequence-based alleles. For instance, researchers have documented overall increases in unique allele numbers by 58.18% [110] and 61.00% [84] compared to length-based polymorphisms alone. Kwon et al. (2021) [111] observed a 2.18-fold increase in the total allele count across 25 autosomal STRs, with a remarkable 4.15-fold increase in SE33, due to the identification of 129 additional unique sequence variations. Numerous studies have shown that the rise in sequence-based allelic diversity significantly boosts key forensic statistical metrics, as evidenced by higher levels of both observed and expected heterozygosity, as well as a notably improved combined power of discrimination [84,110,112,113]. Incorporating sequence variations in both the core repeat and surrounding regions has been shown to significantly reduce the combined random match probability by more than 2.58 × 10^4^ times compared to traditional CE methods, a pattern that was consistently observed across other forensic parameters, attributable to a notable increase in the presence of unique alleles [109]. Ultimately, these data-rich profiles aid in resolving highly challenging forensic tasks. The increased resolution enables investigators to extract multidimensional information from a single profile, thereby significantly improving the deconvolution of complex DNA mixtures by increasing the proportion of fully resolved multiallelic loci and enhancing the statistical confidence required for distant-kinship identification [20,68,84].

Various commercial MPS platforms tailored for forensic applications have been developed for this purpose. Notable examples include the ForenSeq^TM^ DNA Signature Prep kit (Verogen, San Diego, CA, USA), the PowerSeq^TM^ platform (Promega, Madison, WI, USA), and the Precision ID GlobalFiler^TM^ NGS STR Panel v2 (Thermo Fisher Scientific, Waltham, MA, USA). Novel MPS panels have significantly expanded, ranging from targeted identity multiplexes to extensive capture panels, such as the 5422-marker FORCE panel [34]. In a survey, 73% of the European forensic laboratories either already possess or plan to acquire an MPS platform [114], indicating that MPS-based supplementary STR analysis is technically feasible within current forensic laboratory workflows, provided validation requirements are met. To support this growth, the STRAND Working Group was established in 2019 to address the critical issue of standardizing sequence-based STR nomenclature, and various specialized software tools, such as STRinNGS, STRait Razor, and STRsearch, have been developed to interpret MPS data [34].

Despite its considerable analytical advantages, several factors continue to limit the routine implementation of MPS in forensic laboratories. The laboratory procedures required are more complex and time-intensive, necessitating robust automation to ensure high throughput and reproducibility. Thus, the initial investment in instrumentation and computational infrastructure remains substantially higher than that required for CE-based workflows [42,52]. In addition, laboratories must undertake extensive developmental and internal validation studies, establish robust quality assurance procedures, and ensure personnel receive specialized training in sequencing technologies and bioinformatic analysis [39,114]. From an analytical standpoint, the current limitations of MPS read length can impede the reproducible sequencing of certain long, complex STRs [52]. The lack of universal standardization in sequence-based nomenclature and the need to maintain compatibility with existing length-based national DNA databases further complicate implementation. Additionally, the substantial volume of data generated by MPS necessitates the use of powerful bioinformatic tools for accurate alignment of millions of STR sequences, as well as high-capacity servers for secure data storage and management of excess data [21,39]. Consequently, while MPS represents the future of forensic genetics, its widespread adoption is expected to occur progressively rather than replacing established CE methodologies in the short term.

## 7. Validation Frameworks and Statistical Interpretation

Before implementing any STR panel in operational casework, it is imperative that it undergo thorough validation, both technically comprehensive and operationally specific to the laboratory in question [115]. The Scientific Working Group on DNA Analysis Methods (SWGDAM) provides the foundational framework for validation, which is divided into two complementary phases [116]. Developmental validation, typically conducted by the manufacturer, determines critical performance characteristics such as sensitivity, resistance to inhibitors, capability to deconvolute mixtures, stutter behavior, and reproducibility, thereby establishing the published parameters against which laboratory implementations are assessed [116,117]. Internal validation, which must be performed by each implementing laboratory, ensures that the system operates consistently across its specific instruments and workflows and establishes empirically derived interpretation thresholds that may differ significantly from the manufacturer’s defaults [116,117]. The Investigator^®^ HDplex experience underscores the importance of this distinction, as Westen et al. (2012) [98] demonstrated that manufacturer-provided stutter filters were inadequate, necessitating in-house calibration.

For supplementary panels that share loci with existing multiplexes, concordance testing is essential to ensure that genotype calls remain consistent across platforms and can be reliably integrated with profiles from other systems [116,118]. Rigorous genomic characterization of novel loci prior to validation is an absolute prerequisite. The case of the D5S2500 misidentification, where distinct microsatellites were inadvertently targeted under the same locus name by different commercial systems [95], serves as a documented consequence of proceeding without it, and highlights the necessity for a precise definition of chromosomal coordinates, repeat structures, and flanking sequences against a current reference genome assembly such as GRCh38 [21].

The transition to MPS has introduced complexities that validation frameworks must explicitly address. MPS resolves internal sequence variation, producing isoalleles and flanking-region variants that have no equivalents in CE data, necessitating specific validation of analytical software for concordance between platforms [21,118]. The ISFG DNA Commission has established nomenclature requirements for sequence-based STR alleles, with backward compatibility with existing national database profiles as a central requirement, supported by reference resources such as STRSeq and STRidER, which are platforms for the audit and curation of STR data [21,119].

Upon validation of a multiplex system, the forensic interpretation of its profiles requires reliance on robust, population-specific allele-frequency databases and a statistical framework tailored to the marker type and analytical context. Prior to the application of frequency data in casework calculations, it is essential to confirm that loci behave independently through Hardy–Weinberg Equilibrium and linkage disequilibrium testing, prerequisites for the product rule, which permits the multiplication of individual locus probabilities into a combined statistic [118,120]. Any deviations from Hardy–Weinberg Equilibrium require thorough investigation before a locus is utilized in casework, as such deviations may indicate genotyping artifacts, population stratification, or null allele effects, as documented at D2S1360 [94]. When these conditions are satisfied, core forensic metrics, including the Random Match Probability, Power of Discrimination, and Power of Exclusion, can be calculated, with a subpopulation correction (θ) applied to account for regional genetic structure and prevent the overstatement of evidential weight [115,120]. The increasing adoption of probabilistic genotyping, which incorporates quantitative data such as peak heights and degradation patterns, reflects a broader transition from binary threshold models to more statistically nuanced approaches [118]. A distinct approach is required for Y-STRs, as their uniparental inheritance and physical linkage preclude the application of the product rule. Haplotype frequencies must instead be estimated using specialized methods, such as the Discrete Laplace approach or reference-database-supported qualitative statements [35].

A significant challenge associated with extended non-core panels is genetic linkage. Several supplementary STR loci are located on the same chromosomes as markers included in standard forensic multiplexes, creating syntenic locus pairs that may not segregate completely independently [91,98]. Although linkage disequilibrium between such loci is often negligible in population studies involving unrelated individuals, this assumption may not hold in kinship analyses. In pedigree reconstruction, sibship testing and other relationship investigations, physically linked loci may be co-inherited more frequently than expected under complete independence, potentially inflating likelihood ratios if analyzed using the product rule alone [80,98]. Consequently, recombination fractions and genetic distances between syntenic loci should be incorporated into statistical calculations whenever these loci are jointly considered [96,98].

Ensuring that all these requirements are met and transparently reported in casework is not merely a refinement but a prerequisite for the responsible deployment of extended non-core STR panels.

## 8. Implementation Challenges

While non-core STRs offer significant advantages for complex forensic cases, their integration into forensic practice continues to face challenges beyond the validation phase, impacting database infrastructure, population data comprehensiveness, and technical efficiency.

A major unresolved issue is the absence of comprehensive genomic audits for many historically reported non-CODIS STRs. These markers were often developed independently by various laboratories or commercial entities, leading to inconsistent genotypes and interlaboratory confusion [69,95].

The transition from CE to MPS has introduced significant practical challenges, particularly regarding naming conventions. Given the forensic community’s reliance on established systems like CODIS and the National DNA Database (NDNAD), it is essential to ensure backward compatibility between sequence-based profiles and the numerous length-based profiles already present in national databases. It is vital to continue using the current systems without making them obsolete, so the new software and naming systems must effectively convert complex MPS sequence strings into specific, length-based allele designations for this to succeed [42,52].

The lack of population-level frequency data for non-core STRs presents an additional challenge. While core markers have been extensively studied across diverse global populations, there is a notable shortage of sequence-based allele-frequency data for other loci, particularly in underrepresented regions such as Africa and Oceania. Without comprehensive, population-specific reference databases, accurately calculating match probabilities and likelihood ratios becomes infeasible, directly affecting the legal admissibility of these markers [12,88].

On a technical level, CE remains the most widely used technique, but it has specific limitations that continue to affect the performance of supplementary STRs. Incorporating additional markers within a single multiplexed reaction often requires larger amplicon sizes, which are more susceptible to degradation and prone to artifacts [42,84], as discussed in Section 5.

Finally, even with panels exceeding 100 STR loci, there are inherent analytical limitations and still unresolved complex scenarios, as they do not represent a universal solution to all forensic challenges. In kinship analysis, expanded panels can reliably resolve first- and second-degree relationships, but identifying third-degree relatives and beyond remains highly unreliable, often resulting in inconclusive likelihood ratios due to the natural reduction in alleles shared identical-by-descent at increasing degrees of separation. Similarly, reliably deconvoluting highly complex mixtures involving more than five contributors remains profoundly difficult regardless of panel size, representing an unresolved limitation of current supplementary STR approaches [20,84].

## 9. Ethical and Legal Considerations

Traditional STR profiling, which relies on CE, has historically operated within well-defined legal parameters. However, the incorporation of non-core STRs into forensic multiplexes and the transition to MPS introduce ethical and legal challenges that extend beyond technical considerations.

The European General Data Protection Regulation categorizes genomic data as highly sensitive, thereby imposing strict restrictions on its acquisition, management, and distribution [121]. The 1997 Council of Europe’s Convention on Biomedicine and Human Rights explicitly safeguards the right to genetic privacy, prohibiting genetic discrimination and the use of clinically predictive information for identification purposes [122]. On a national level, individual countries can establish specific legal frameworks. In Portugal, Law No. 5/2008 sets out explicit rules for the collection, storage, and destruction of forensic DNA profiles, emphasizing the importance of informed consent and the proportionality of genetic data use, balancing investigative needs with privacy protections [123].

In this same note, the core ethical principle of forensic DNA typing is that STR profiles serve solely as identity markers, acting as neutral identifiers without revealing sensitive medical or phenotypic information [3,4]. Importantly, a distinction must be made between the use of supplementary STRs for human identification and kinship analysis and the use of other genomic markers for ancestry inference, forensic DNA phenotyping, or forensic investigative genetic genealogy. Supplementary STR panels remain primarily identity-focused tools that provide additional discriminatory power without intentionally generating predictive information about an individual’s appearance, ancestry, or health status [4]. By contrast, ancestry-informative, phenotype-informative and genealogy-based approaches involve substantially broader privacy considerations and therefore require distinct ethical oversight, legal safeguards, and governance frameworks consistent with the principle of proportionality [124].

Nonetheless, emerging evidence indicates that non-coding STRs may influence gene expression markers, such as TH01, in relation to schizophrenia, though causal relationships remain unestablished [3]. This area, although widely debated, necessitates careful consideration when selecting these markers, such as excluding clinically significant variants, as failing to do so constitutes a violation of human rights [4]. Furthermore, the linkage disequilibrium between forensic STRs and nearby SNPs could inadvertently reveal biogeographical ancestry or disease susceptibilities [124]. These findings challenge the legal notion that forensic DNA profiles disclose identity alone, raising questions about genetic privacy.

The primary concern lies not in the markers themselves, but in the broader applications enabled by expanded genomic panels. For instance, Forensic DNA Phenotyping (FDP) and biogeographical ancestry inference offer investigative value when no database match is found, but they also carry risks of interpretive errors and ethnic profiling, which can mislead investigations and unfairly target minority groups [4,124]. The use of high-density panels may also be advantageous in Forensic Investigative Genetic Genealogy (FIGG), which leverages consumer DNA databases to identify suspects through distant familial matches, but it presents a unique issue, as it can blur the lines of informed consent and data ownership [125].

Another concern is the generation of massive volumes of data by MPS, frequently exceeding the requirements of the specific forensic question at hand. This creates the need for stringent bioinformatic management to ensure effective data filtering and raw data handling, mitigating the risk of any data breaches [22]. In all these areas, implementation is progressing faster than legislation.

While standard STR testing adheres to strict admissibility criteria, the use of expanded non-core panels and sequencing technologies remains largely unregulated by specific legal frameworks in most regions. Establishing governance structures that define the permissible scope of phenotypic inference, require informed consent for database inclusion, and ensure robust protection of the vast amount of sensitive genomic data is crucial for maintaining both scientific integrity and public trust in forensic DNA evidence [3,4,124,125].

## 10. Future Perspectives

To fully harness the investigative potential of non-core STRs and MPS technologies, the field must adopt a highly interdisciplinary and collaborative approach across genomics, bioinformatics, population science, and legal governance.

As MPS gradually establishes its role, a primary objective is to comprehensively define all aspects related to it. It is essential to conduct thorough genomic audits of all genetic markers in a unified, internationally recognized manner and to gradually expand existing national DNA databases to include both traditional length-based profiles and high-resolution sequence data [21,64]. Importantly, this expansion should be accompanied by efforts to enhance the representation of currently underrepresented populations [35,88].

Future multiplexes are anticipated to evolve towards integrated “all-in-one” panels that combine additional STRs with alternative marker types, such as SNPs, InDels, and microhaplotypes, becoming even more informative and reducing artifact burden, thereby improving data recovery [42,107]. Innovations in hardware, including mini microfluidic devices and advancements in nanopore sequencing, have the potential to facilitate rapid, on-site genetic analysis directly at crime scenes, offering swift STR typing by integrating all processing steps into a single portable device [126,127].

Beyond genetics, the field is advancing towards non-targeted multi-omics approaches that integrate genetic ancestry inference with epigenetic DNA methylation profiling, RNA analysis, and microbiome characterization, allowing for the simultaneous estimation of biogeographical origin, chronological age, and tissue source from a single forensic sample [5,22].

Managing the unprecedented volume and complexity of data generated by expanded genomic panels will necessitate the development of robust, user-friendly bioinformatic pipelines. The integration of artificial intelligence and machine learning algorithms holds significant promise for aiding interpretation, automating complex tasks, uncovering hidden data patterns, and facilitating complex mixture deconvolution [128,129]. These technologies can also be applied to enhance Forensic DNA Phenotyping (FDP) and Forensic Investigative Genetic Genealogy (FIGG) algorithms [1].

As forensic methodologies delve deeper into genomic information, the transnational legal and ethical frameworks governing their use must be continuously updated to keep pace. Ensuring global inclusivity in population database development and establishing governance structures that protect genetic privacy while enabling the responsible use of advanced forensic tools will be essential to maintaining both scientific integrity and public trust [4].

## 11. Conclusions

This review provides a comprehensive examination of genetic markers in human identification, emphasizing the scientific foundation, practical applications, and broader implications of incorporating additional non-core STR loci into forensic practice, alongside the transition to MPS. The evidence indicates that non-core STRs, when rigorously validated and supported by robust population databases, offer a scientifically valid solution to the limitations of standard core panels, providing significantly enhanced discriminatory power for complex kinship analysis, degraded DNA typing, and mixture deconvolution.

However, this increased capability is accompanied by unresolved challenges in genomic characterization, nomenclature standardization, database compatibility, statistical methodology, and ethical governance, which must be systematically addressed.

Practically, a hybrid approach that maintains CE-based profiling for routine, high-throughput casework while selectively employing MPS and extended non-core marker panels for complex scenarios appears to be the most operationally feasible path forward for forensic laboratories operating under current resource and regulatory constraints. From an operational perspective, implementation of such a strategy could follow a phased approach. Conventional CE-based STR profiling would remain the primary method for routine casework, while supplementary non-core STR panels and MPS technologies would be selectively applied to cases involving degraded DNA, complex mixtures, distant kinship investigations or other challenging evidential scenarios. Such a model would allow laboratories to progressively develop validation frameworks, bioinformatic expertise and population databases while minimizing disruption to established forensic workflows. From a practical implementation perspective, future efforts should prioritize five key areas: (i) comprehensive internal validation of supplementary STR panels before routine implementation; (ii) expansion of population reference databases, particularly for underrepresented populations; (iii) harmonization of sequence-based nomenclature to facilitate interoperability and database compatibility; (iv) systematic genomic auditing of forensic markers to ensure accurate locus characterization; and (v) development of evidence-based guidelines defining the circumstances in which supplementary STRs provide meaningful advantages in complex kinship investigations, degraded DNA analysis and mixture interpretation. Particular attention should be given to the development of population databases for currently underrepresented regions, including African [130] and Middle Eastern populations, to ensure equitable and statistically robust forensic applications across diverse population groups. Addressing these priorities will be essential for the responsible and sustainable integration of supplementary STRs into routine forensic practice.

## Figures and Tables

**Table 1:** Overview of the main genetic marker classes used in forensic genetics ^1^.

Marker	Amplicon Size	Mutation Rate	Discrimination Power	Main Forensic Application	Main Forensic Limitation
A-STR	100–400 bp	Very High	Very High	Routine identification and kinship; Databases	Limited in degraded samples; Stutter artifacts
Y-STR	100–400 bp	Variable (Higher for RM-YSTRs)	High for lineage; Low for individualization (Except RM-YSTRs)	Resolving male/female mixtures like in sexual crimes; Paternal lineage tracing	Lacks recombination and cannot individualize males (Only by RM-YSTRs)
X-STR	100–400 bp	High	High	Complex deficiency kinship; cases involving females	Uninformative for father–son testing; Requires complex statistics
mtDNA	<200 bp or mitogenome	Stable over generations	High for lineage; Low for individualization	Ancient/degraded samples; Mass disasters; Maternal lineage	Cannot individualize; Heteroplasmy; Complex analysis
SNP	50–150 bp	Low	Low per locus	Degraded samples; Characteristics prediction; Lineage tracing	Requires large MPS panels; Poor for mixture deconvolution
InDel	60–160 bp	Low	Low per locus	Degraded Samples; Population admixture; Anthropology	Requires large panels; Poor for mixture deconvolution
MH	<300 bp, often <100 bp	Low	High	Mixture deconvolution; Ancestry inference; Kinship; Individualization	Reliance on MPS; Lack of database information

^1^ Data compiled and adapted from [7,9,12,15,23,24,25,26,27,28,29,30,31,32,33,34]. Abbreviations: bp, base pairs; A-STR, autosomal short tandem repeat; Y-STR, Y-chromosomal short tandem repeat; RM-YSTR, rapidly mutating Y-chromosomal short tandem repeat; X-STR, X-chromosomal short tandem repeat; mtDNA, mitochondrial DNA; SNP, single-nucleotide polymorphism; InDel, insertion/deletion polymorphism; MH, microhaplotype; MPS, massively parallel sequencing.

**Table 2:** Overview of loci included in the Investigator^®^ HDplex kit ^1^.

Locus	Core Status	Location	Repeat Motif	Reported Artifacts
D12S391	CODIS/ESS	12p13.2	Compound Tetranucleotide	Frequently exhibits high minus stutter ratios [61,92].
D18S51	CODIS/ESS	18q21.33	Simple Tetranucleotide	Deletions (2 bp) in 3′-flanking regions can produce microvariant alleles [12].
SE33	Non-core (Very common in other kits)	6q14	Complex Tetranucleotide	Large allele range and high average stutter ratio [12,93].
D2S1360	Non-core	2p24-p22	Complex Tetranucleotide	Point mutation (G > A) in the primer binding site can cause heterozygote peak imbalances and allele dropouts [94].
D3S1744	Non-core	3q24	Complex Tetranucleotide	Susceptible to off-ladder microvariant alleles [82]
D4S2366	Non-core	4p16-p15.2	Complex Tetranucleotide	Departures from Hardy–Weinberg equilibrium in some groups, such as the North Italian [76]
D5S2500	Non-core	5q11.2	Complex Tetranucleotide	Documented misidentification, with locus name ambiguity (correct in Investigator^®^ HDplex) [95]
D6S474	Non-core	6q21-22	Complex Tetranucleotide	Alleles may amplify as 1-repeat less [80]
D7S1517	Non-core	7q31.3	Compound	Single-base-pair length variants and rare triallelic patterns [86]
D8S1132	Non-core	8q23.1	Complex Tetranucleotide	Generally stable but exhibits typical minus stuttering [93]
D10S2325	Non-core	10p12	Simple Pentanucleotide	~0.5 nt peak shift from the ladder; artifactual plus stutters; that migrates into SE33 range and uneven triallelic patterns [85,86,91]
D21S2055	Non-core	21q22	Complex/Compound	Peak height imbalances for heterozygous; Extremely off-ladder alleles and stutters; uneven triallelic patterns [91,96,97].

^1^ Data compiled and adapted from [12,61,68,75,76,79,80,82,85,86,91,92,93,94,95,96,97,98].

**Table 3:** Overview of population-based studies using the Investigator^®^ HDplex kit.

Population/Region	Sample Size	Main Findings	References
HGDP-CEPH worldwide panel	941	High global informativeness; differentiation among continental groups	[67]
Dutch population	335	High forensic efficiency; technical artifacts characterized	[98]
North Italian population	359	High combined power of discrimination and exclusion	[76]
Polish population	303	High polymorphism and forensic usefulness	[77]
Swiss population	1198	Strong forensic parameters across Investigator^®^ HDplex loci	[78]
Portuguese population	176	High polymorphism in the Lisbon population	[99]
South Korean population	990	High discrimination; population-specific allele frequencies established	[86]
Japanese population	344	Informative loci; locus-specific artifacts reported	[85]
Chinese Han populations	183/503	Strong supplementary value when combined with standard kits	[81,82]
Argentine population	980	Reference database established; regional genetic patterns assessed	[91]
Ecuadorian population	200	High informativeness for forensic purposes	[100]
Russian population	558	High forensic efficiency; SE33 highly polymorphic	[101]

**Table 4:** Syntenic STR locus pairs to consider when combining Investigator^®^ HDplex markers with standard CODIS/ESS STR multiplexes ^1^.

Chromosome	Investigator^®^ HDplex Locus	CODIS/ESS Locus
Chr 2	D2S1360	TPOX
		D2S441
		D2S1338
Chr 3	D3S1744	D3S1358
Chr 4	D4S2366	FGA
Chr 5	D5S2500	CSF1PO
		D5S818
Chr 7	D7S1517	D7S820
Chr 8	D8S1132	D8S1179
Chr 10	D10S2325	D10S1248
Chr 12	D12S391	vWA
Chr 21	D21S2055	D21S11

^1^ Data compiled and adapted from [91,96,98,106].

## Data Availability

No new data were created or analyzed in this study. Data sharing is not applicable to this article.

## Abbreviations

The following abbreviations are used in this manuscript:

A-STR	Autosomal Short Tandem Repeat
aiSNP	Ancestry Informative Single-Nucleotide Polymorphism
bp	Base Pairs
CE	Capillary Electrophoresis
CEPH	Centre d’Etude du Polymorphisme Humain
CODIS	Combined DNA Index System
DIP-SNP	Deletion/Insertion Polymorphism linked to SNP
DIP-STR	Deletion/Insertion Polymorphism linked to STR
DNA	Deoxyribonucleic Acid
EDNAP	European DNA Profiling Group
ENFSI	European Network of Forensic Science Institutes
ESS	European Standard Set
FBI	Federal Bureau of Investigation
FDP	Forensic DNA Phenotyping
FGA	Fibrinogen Alpha Chain (locus)
FIGG	Forensic Investigative Genetic Genealogy
GRCh38	Genome Reference Consortium Human Build 38
HGDP	Human Genome Diversity Project
iiSNP	Identity Informative Single-Nucleotide Polymorphism
InDel	Insertion/Deletion Polymorphism
ISFG	International Society for Forensic Genetics
MH	Microhaplotype
MPS	Massively Parallel Sequencing
mtDNA	Mitochondrial DNA
NDIS	National DNA Index System
NDNAD	National DNA Database
NGS	Next-Generation Sequencing
PCR	Polymerase Chain Reaction
piSNP	Phenotype Informative Single-Nucleotide Polymorphism
RFLP	Restriction Fragment Length Polymorphism
RM-YSTRs	Rapidly mutating Y-STRs
RNA	Ribonucleic Acid
SNP	Single-Nucleotide Polymorphism
STR	Short Tandem Repeat
STRidER	STR allele frequency database and reference resource
STRSeq	STR sequence database resource
STRAND	Sequencing-based STR Allele Nomenclature Working Group
SWGDAM	Scientific Working Group on DNA Analysis Methods
TH01	Tyrosine Hydroxylase 1 (locus)
VNTR	Variable Number Tandem Repeat
vWA	von Willebrand Factor A (locus)
X-STR	X Chromosome Short Tandem Repeat
Y-STR	Y Chromosome Short Tandem Repeat
